# Novel immune-related prognostic model and nomogram for breast cancer based on ssGSEA

**DOI:** 10.3389/fgene.2022.957675

**Published:** 2023-01-10

**Authors:** Linrong Li, Lin Li, Mohan Liu, Yan Li, Qiang Sun

**Affiliations:** ^1^ Department of Breast Surgery, Peking Union Medical College Hospital, Peking Union Medical College, Chinese Academy of Medical Sciences, Beijing, China; ^2^ Department of Joint and Orthopedics, Zhujiang Hospital, Second Clinical Medical College, Southern Medical University, Guangzhou, China

**Keywords:** immune, prognostic, model, breast cancer, ssGSEA, nomogram

## Abstract

This study aimed to construct an immune-related prognostic model and a nomogram to predict the 1-, 3-, and 5-year overall survival (OS) of breast cancer patients. We applied single-sample gene set enrichment analysis to classify 1,053 breast cancer samples from The Cancer Genome Atlas (TCGA) database into high and low immune cell infiltration clusters. In cluster construction and validation, the R packages “GSVA,” “hclust,” “ESTIMATE,” and “CIBERSORT” and GSEA software were utilized. ImmPort, univariate Cox regression analysis, and Venn analysis were then used to identify 42 prognostic immune-related genes. Eventually, the genes *TAPBPL*, *RAC2*, *IL27RA*, *ULBP2*, *PSMB8*, *SOCS3*, *NFKBIE*, *IGLV6-57*, *CXCL1*, *IGHD*, *AIMP1*, and *CXCL13* were chosen for model construction utilizing least absolute shrinkage and selection operator regression analysis. The Kaplan–Meier curves of both the training and validation sets indicated that the overall survival of patients in the low-risk group was superior to that of patients in the high-risk group (*p* < .05). The areas under curves (AUCs) of the model at 1, 3, and 5 years were, respectively, .697, .710, and .675 for the training set and .930, .688, and .712 for the validation set. Regarding clinicopathologic characteristics, breast cancer-related genes, and tumor mutational burden, effective differentiation was achieved between high-risk and low-risk groups. A nomogram integrating the risk model and clinicopathologic factors was constructed using the “rms” R software package. The nomogram’s 1-, 3-, and 5-year AUCs were .828, .783, and .751, respectively. Overall, our study developed an immune-related model and a nomogram that could reliably predict OS for breast cancer patients, and offered insights into tumor immune and pathological mechanisms.

## 1 Introduction

Breast cancer is one of the most prevalent cancers affecting women worldwide. According to the American Cancer Society, one in eight women in the United States will be diagnosed with invasive breast cancer during their lifetime, and 1 in 39 will eventually die from the disease (DeSantis et al., 2019). Based on the estrogen receptor (ER) or progesterone receptor expression status and human epidermal growth factor 2 (HER2) gene amplification, breast cancer is divided into three major subgroups: hormone receptor+/HER2–, HER2+, and triple-negative breast cancer (TNBC) (Waks and Winer, 2019). Over the past 30 years, breast cancer patients’ 5-year relative survival rate has increased to 83%–92% [Surveillance, Epidemiology, and End Results (SEER) Program (https://www.seer.cancer.gov/), Seer, 2019]. However, breast cancer is a molecularly heterogeneous disease in which the treatment and outcomes vary significantly between subgroups (Waks and Winer, 2019). Therefore, it is imperative that prognostic factors associated with the biological heterogeneity of breast cancer be identified in order to improve survival.

In recent decades, mounting evidence has indicated that breast cancer is characterized by its immune landscape (Nagarajan and McArdle, 2018). Numerous studies revealed that the survival time of breast cancer patients positively correlated with tumor-infiltrating lymphocytes (TILs), particularly in HER2+ and TNBC subtypes (Loi, 2013; Adams et al., 2014). Following the promising clinical outcomes of cancer immunotherapies, interest in the field of immune microenvironment has increased (Waldman et al., 2020). In phase 3 studies on TNBC, programmed cell death ligand 1 (PD-L1) inhibitor atezolizumab and programmed cell death 1 (PD-1) inhibitor pembrolizumab have been evaluated. In the phase 3 IMpassion130 trial, the addition of atezolizumab to nab-paclitaxel as frontline therapy for patients with unresectable and advanced TNBC improved the survival rate (Schmid et al., 2020b). Pembrolizumab plus neoadjuvant chemotherapy significantly increased the pathological complete response rate in patients with untreated early-stage TNBC in the phase 3 study (NCT03036488) (64.8% *versus* 51.2%, *p* < .001) (Schmid et al., 2020a). Therefore, immune-related biomarkers of the tumor immune microenvironment may be used as prognostic indicators in breast cancer.

**FIGURE 1:**
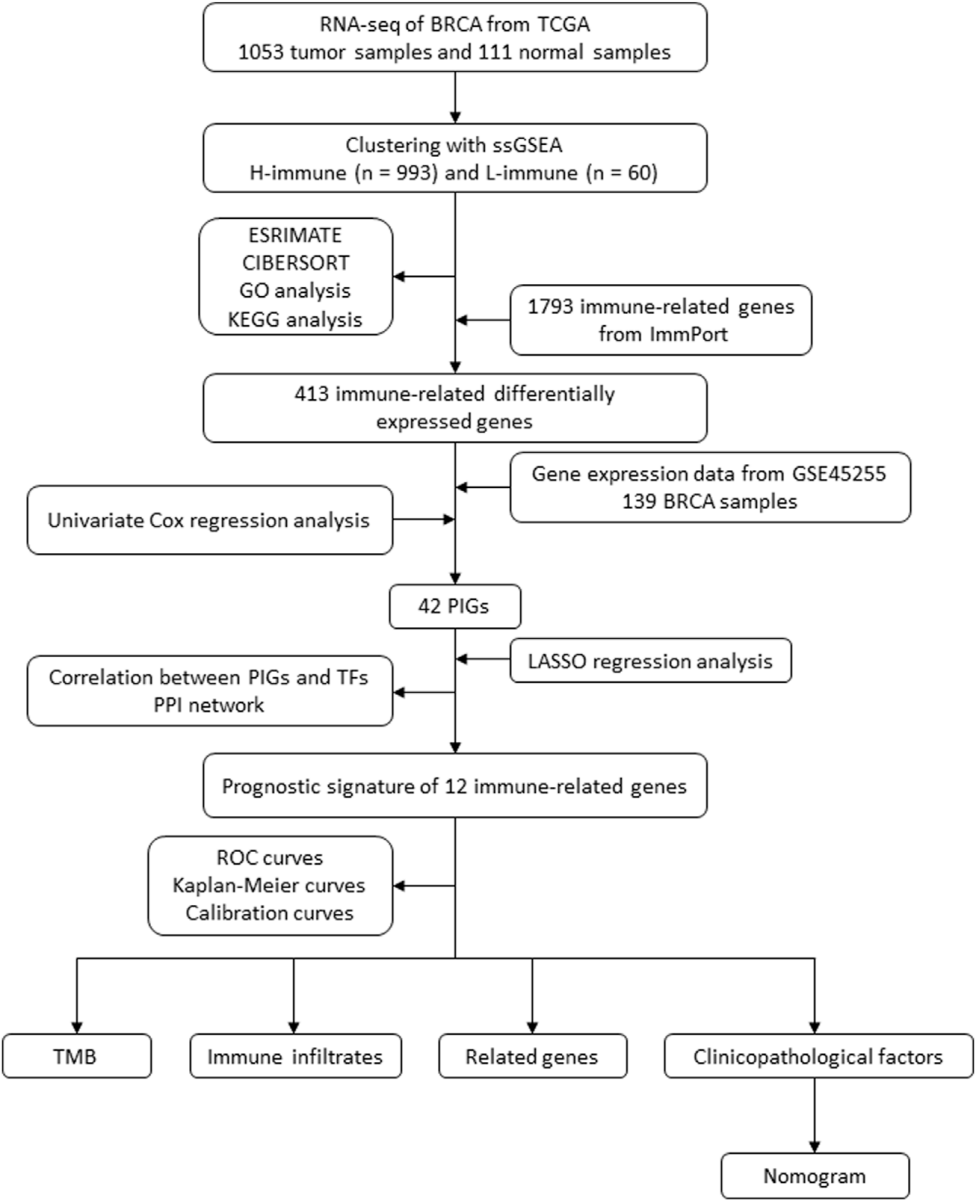
Flow diagram of the study. BRCA, breast cancer; TCGA, The Cancer Genome Atlas; ssGSEA, single-sample gene set enrichment analysis; KEGG, Kyoto Encyclopedia of Genes and Genomes; GO, Gene Ontology; PIG, prognostic immune-related gene; LASSO, least absolute shrinkage and selection operator; TF, transcription factor; PPI, protein–protein interaction; ROC, receiver operating characteristic; TMB, tumor mutational burden.

By examining the immune landscape of breast cancer, we aimed to develop a robust prognostic signature. After dividing breast cancer samples into high and low immune cell infiltration clusters using single-sample gene set enrichment analysis (ssGSEA), 413 immune-related differentially expressed genes (DEGs) between clusters were identified using ImmPort, and 12 prognostic immune-related DEGs were selected using Cox and least absolute shrinkage and selection operator (LASSO) regression analyses. The model correlated with clinicopathologic factors, immune infiltrates, genes associated with breast cancer, and tumor mutational burden (TMB). Finally, we successfully developed a nomogram to predict the 1-, 3-, and 5-year overall survival (OS) of breast cancer patients (Figure 1).

## 2 Materials and methods

### 2.1 Construction and validation of immune clustering

The RNA sequencing data and clinical information on breast cancer patients were obtained from The Cancer Genome Atlas (TCGA, https://cancergenome.nih.gov/) database. This study included patients with ductal and lobular breast cancer but excluded male patients. The original data were summarized and processed with Strawberry Perl v5.0.1. R version 4.1.1 was utilized for data analysis (R Core Team, 2019). The ssGSEA was performed on each breast cancer sample in TCGA database based on the expression of 29 immune cell types using the “GSVA” R package. Using the unsupervised hierarchical clustering function “hclust” of R software, breast cancer samples were divided into clusters with high and low immune cell infiltration based on immune infiltration levels. The hierarchical relationship between samples was depicted using a dendrogram generated by the “sparcl” R package.

To validate the clustering results, we compared the immune status of the two clusters of immune cell infiltration using two R software algorithms. Based on specific gene expression levels, the “ESTIMATE” algorithm of R software was used to calculate the Immune Score, Stromal Score, ESTIMATE Score, and tumor purity of each sample (Yoshihara et al., 2013). Next, we utilized the “CIBERSORT” R software package to compare the expression levels of human leukocyte antigen (HLA) family genes and the proportion of 22 immune cell types between the two clusters (Newman et al., 2015). The R package “ggpubr” illustrated the clustering heatmap, violin plots, and boxplots.

### 2.2 Gene set enrichment analysis

Using GSEA (version 4.1.0), gene set enrichment analysis was performed between clusters of high and low immune cell infiltration in TCGA database (Mootha et al., 2003; Subramanian et al., 2005). We used the gene expression data from TCGA database to perform KEGG pathway analysis and Gene Ontology (GO) functional annotations to identify enriched molecular mechanisms and cellular functions in the cluster with high immune cell infiltration. False discovery rate (FDR) values < .01 were considered statistically significant. The R packages “reshape” and “ggplot2” were used to create bubble charts.

### 2.3 Identification of differentially expressed immune-related genes between clusters

DEGs were identified through differential gene expression analysis of samples from the two clusters in TCGA database. Significant stipulations were |LogFC| > .585 and FDR <.05. Immune-related genes were obtained from the Immunology Database and Analysis Portal (ImmPort, https://www.immport.org/) (Bhattacharya et al., 2018). We utilized Venn analysis to identify immune-related DEGs between high and low immune cell infiltration clusters in breast cancer. The R packages “limma,” “ggplot2,” “venn,” and “heatmap” were utilized.

### 2.4 Construction of functional interaction networks of prognostic immune-related proteins and transcription factors

Gene expression profiles and clinical details of GSE45255 (GPL96 platform part) were downloaded from the Gene Expression Omnibus (GEO, https://www.ncbi.nlm.nih.gov/geo/) database (Nagalla et al., 2013). Based on immune-related DEGs and clinical data from the TCGA database, univariate Cox regression analysis was conducted to identify prognostic immune-related genes (PIGs) shared by TCGA and GSE45255 databases significantly correlated with the OS of breast cancer patients, with *p* < .01 considered statistically significant. Next, we conducted the co-expression analysis of PIGs and transcription factors (TFs) downloaded from the Cistrome platform (https://www.cistrome.org/) to identify PIG-related TFs and TF-related PIGs. The significant thresholds were determined to be |cor| > .4 and FDR <.001, and the R software packages “ggalluvial,” “ggplot2,” and “dplyr” were utilized in the procedure. In addition, to illustrate potential interactions between PIGs and TFs, we performed protein–protein interaction (PPI) network analysis using the online Search Tool for the Retrieval of Interacting Genes/Proteins (STRING, https://string-db.org) (Damian et al., 2021). The confidence score threshold was set to .7 (high), and disconnected network nodes were hidden.

### 2.5 Construction and validation of an immune-related prognostic model

Based on the identified PIGs, we performed LASSO regression analysis to screen out genes for model construction using the “glmnet” algorithm. In the prognostic model, each patient was assigned a risk score based on the following formula:

$$RiskScore=\overset{n}{\underset{i=1}{\sum }}Coe\;PIG_{i}\times \mathrm{Exp}\;PIG_{i}.$$

According to the median risk score, patients were divided into high- and low-risk groups. TCGA and GSE45255 samples were used as training and validation sets, respectively. The R software packages “survival,” “survminer,” “timeROC,” and “rms” were used to plot Kaplan–Meier (K–M) curves, time-dependent receiver operating characteristic (ROC) curves, and calibration curves. Next, univariate and multivariate Cox regression analyses with a significant value of *p* < .05 were conducted to determine whether the model was an independent prognostic factor for breast cancer.

### 2.6 Correlation analysis between PIGs and immune infiltrates

We examined correlations between PIGs in the model and immune infiltrates using the “CIBERSORT” algorithm, where *p* < .05 was considered statistically significant. R software packages “reshape2,” “tidyverse,” and “ggplot2” were used to illustrate the results.

### 2.7 Correlation analysis between cancer-related genes and the prognostic model

To identify potential associations between the prognostic model and the expression level of breast cancer-related genes, we performed correlation analysis of BRCA1 (breast cancer 1), BRCA2 (breast cancer 2), PDCD1 (programmed cell death 1), and CTLA4 (cytotoxic T-lymphocyte-associated protein 4) with the risk score and risk group. The R software packages “limma” and “ggpubr” were utilized in the process.

### 2.8 TMB analysis

The TMB data on breast cancer were downloaded from TCGA database’s simple nucleotide variation section. Differential analysis of TMB was performed on breast cancer samples with high and low risks. To investigate correlations between TMB and prognosis, we divided breast cancer samples from TCGA database into high- and low-TMB groups, using a TMB cutoff value of one mutation (mut) per megabase (MB). Survival analysis was conducted on different TMB and risk-score groups, and Kaplan–Meier survival curves were plotted utilizing the “survival” and “survminer” R software packages.

### 2.9 Construction and validation of a nomogram

We built a nomogram to predict the survival of breast cancer patients using the “rms” and “survival” R packages. Initially, the T stage, N stage, M stage, clinical stage, risk group, and age were considered variables for constructing the nomogram. In order to evaluate the prognostic value of the nomogram, the ROC curve and calibration curve were drawn.

## 3 Results

### 3.1 Construction and validation of immune clustering

**FIGURE 2:**
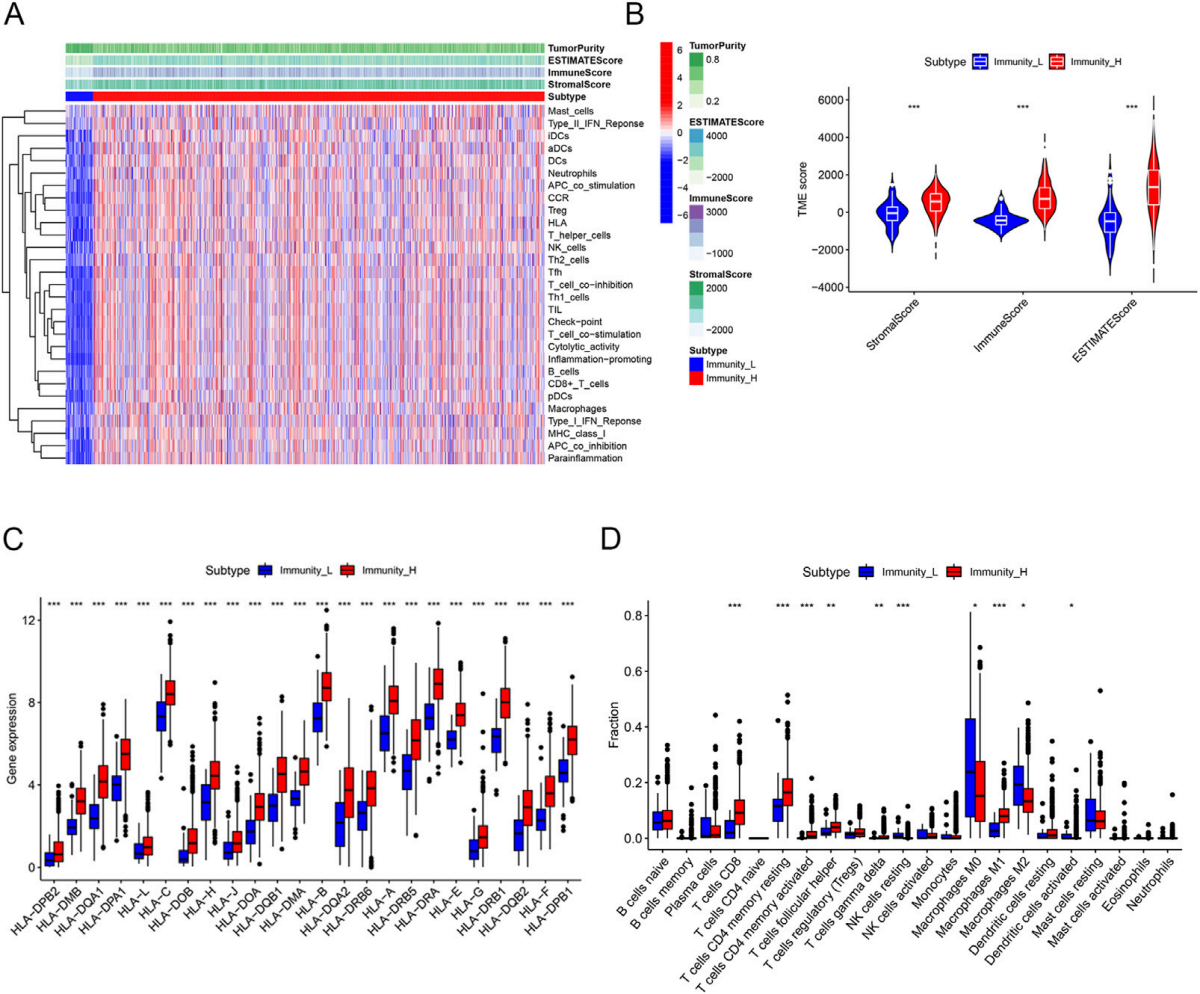
Construction and validation of immune clustering. **(A)** Heatmap showing enrichment levels of 29 immune cell types between the two clusters. The Immune Score, Stromal Score, ESTIMATE Score, and tumor purity can be seen on top of the genes. **(B)** Violin plots showing the Stromal Score, Immune Score, and ESTIMATE Score of the two clusters. **(C)** Boxplots showing the gene expression of the HLA family of the two clusters. **(D)** Boxplots showing proportions of 22 immune cell types between the two clusters. Proportions of CD8 T cells, resting memory CD4 T cells, CD4 memory activated T cells, follicular helper T cells, gamma delta T cells, and M1 macrophages were higher in the cluster with high immune cell infiltration, whereas the proportions of resting NK cells, M0 macrophages, M2 macrophages, and activated dendritic cells were lower in the cluster with high immune cell infiltration.

RNA sequencing data involving 39,740 mRNAs from a total of 1,053 breast cancer samples and 111 normal samples were obtained from TCGA database, along with the clinical data on 1,053 breast cancer patients. We obtained the ssGSEA immune enrichment score for each breast cancer sample. Based on the infiltration levels of 29 immune cell types, clusters of breast cancer samples with high (*n* = 993) and low (n = 60) immune cell infiltration were identified (Figure 2A). The Immune Score, Stromal Score, ESTIMATE Score, and tumor purity were calculated using the “ESTIMATE” algorithm in order to validate the clustering. The cluster with low immune cell infiltration exhibited lower Immune Score, Stromal Score, ESTIMATE Score, and tumor purity than the cluster with high immune cell infiltration (Figures 2A,B). In addition, the “CIBERSORT” algorithm determined that the cluster with high immune cell infiltration had higher expression levels of all HLA subtypes and higher proportions of 6 out of 22 immune cell types than the cluster with low immune cell infiltration (Figures 2C,D).

### 3.2 Gene set enrichment analysis

**FIGURE 3:**
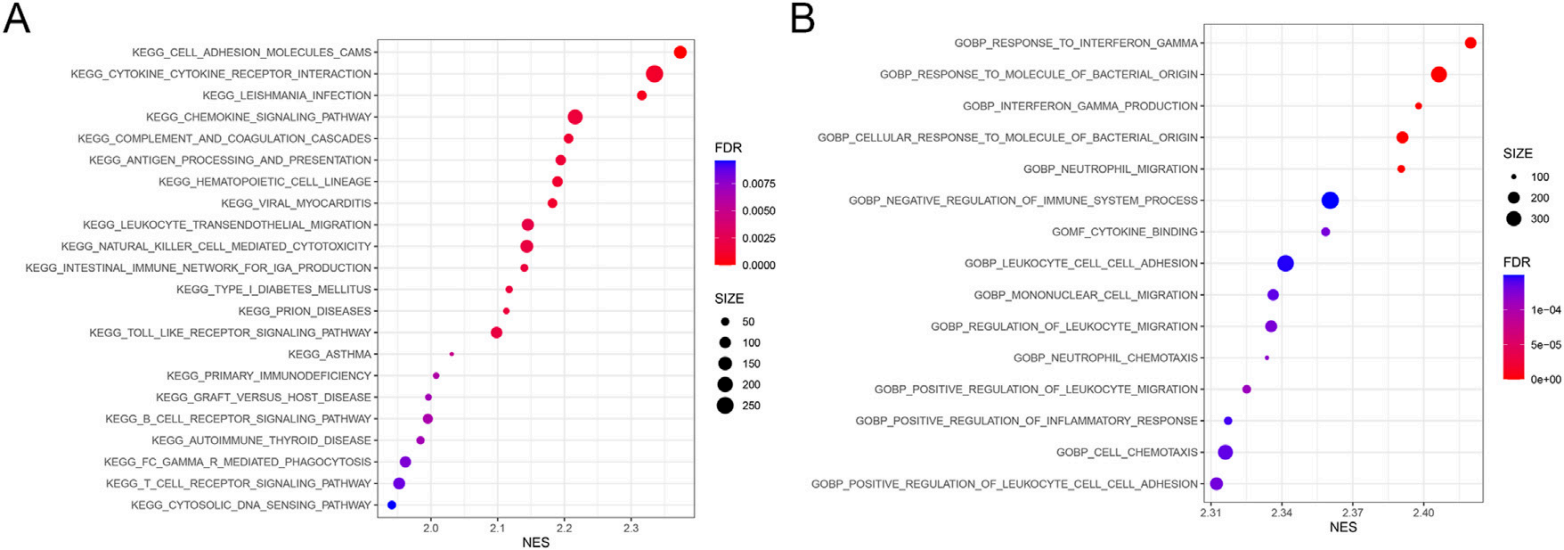
Results from gene set enrichment analysis. The size and color of the bubble represent the number of genes enriched in the pathway/function and the statistical significance, respectively. **(A)** 22 KEGG pathways. **(B)** Top 15 GO cellular functions. FDR, false discovery rate; NES, normalized enrichment score.

Gene set enrichment analysis was conducted within clusters with high and low immune cell infiltration. KEGG pathway results demonstrated that the DEGs of the high immune cell infiltration cluster were mainly involved in the signaling pathways of cytokine receptor interaction, chemokine, natural killer cell-mediated cytotoxicity, and toll-like receptor (Figure 3A). GO analysis demonstrated that the DEGs in the cluster with high immune cell infiltration were significantly associated with the response to the molecule of bacterial origin (Figure 3B).

### 3.3 Identification of differentially expressed immune-related genes between clusters

**FIGURE 4:**
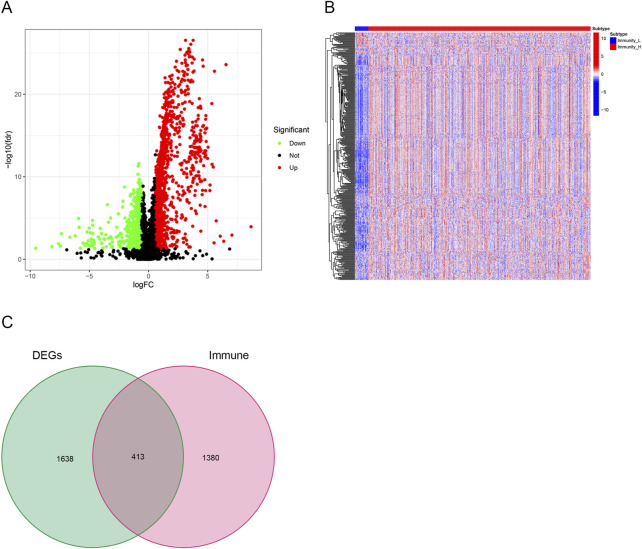
Identification of differentially expressed immune-related genes. **(A)** Volcano plots showing 845 downregulated genes (green) and 1,206 upregulated genes (red) in the cluster with high immune cell infiltration. **(B)** Heatmaps showing 27 downregulated immune-related genes (blue) and 386 upregulated immune-related genes (red) in the cluster with high immune cell infiltration. **(C)** Venn diagram showing the intersection of differentially expressed genes (DEGs) and immune-related genes. In all, 413 overlapped genes were identified.

Using a threshold of |LogFC| > .585 and FDR <.05, 2051 DEGs were obtained from TCGA database between high and low immune cell infiltration clusters. A total of 845 genes were downregulated and 1,206 genes were upregulated in the cluster with high immune cell infiltration (Figure 4A). In addition, a list of 1,793 immune-related genes was obtained from ImmPort. A total of 413 immune-related DEGs were identified through Venn analysis of two sets of genes, containing 27 downregulated genes and 386 upregulated genes in the cluster with high immune cell infiltration (Figures 4B,C).

### 3.4 Construction of functional interaction networks of prognostic immune-related proteins and TFs

**FIGURE 5:**
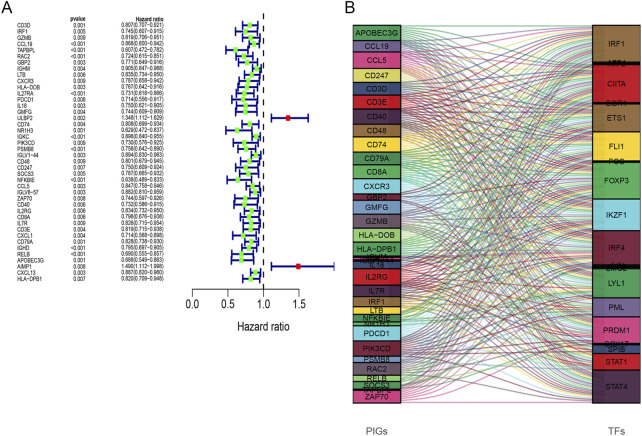
**(A)** Forest plots of hazard ratios for overall survival of 42 prognostic immune-related genes. **(B)** Sankey diagram illustrating the relationship between 35 prognostic immune-related genes (left) and 21 transcription factors (right) for the same plot. The box height indicates the percentage of TF and PIG varieties, while lines show the links.

**FIGURE 6:**
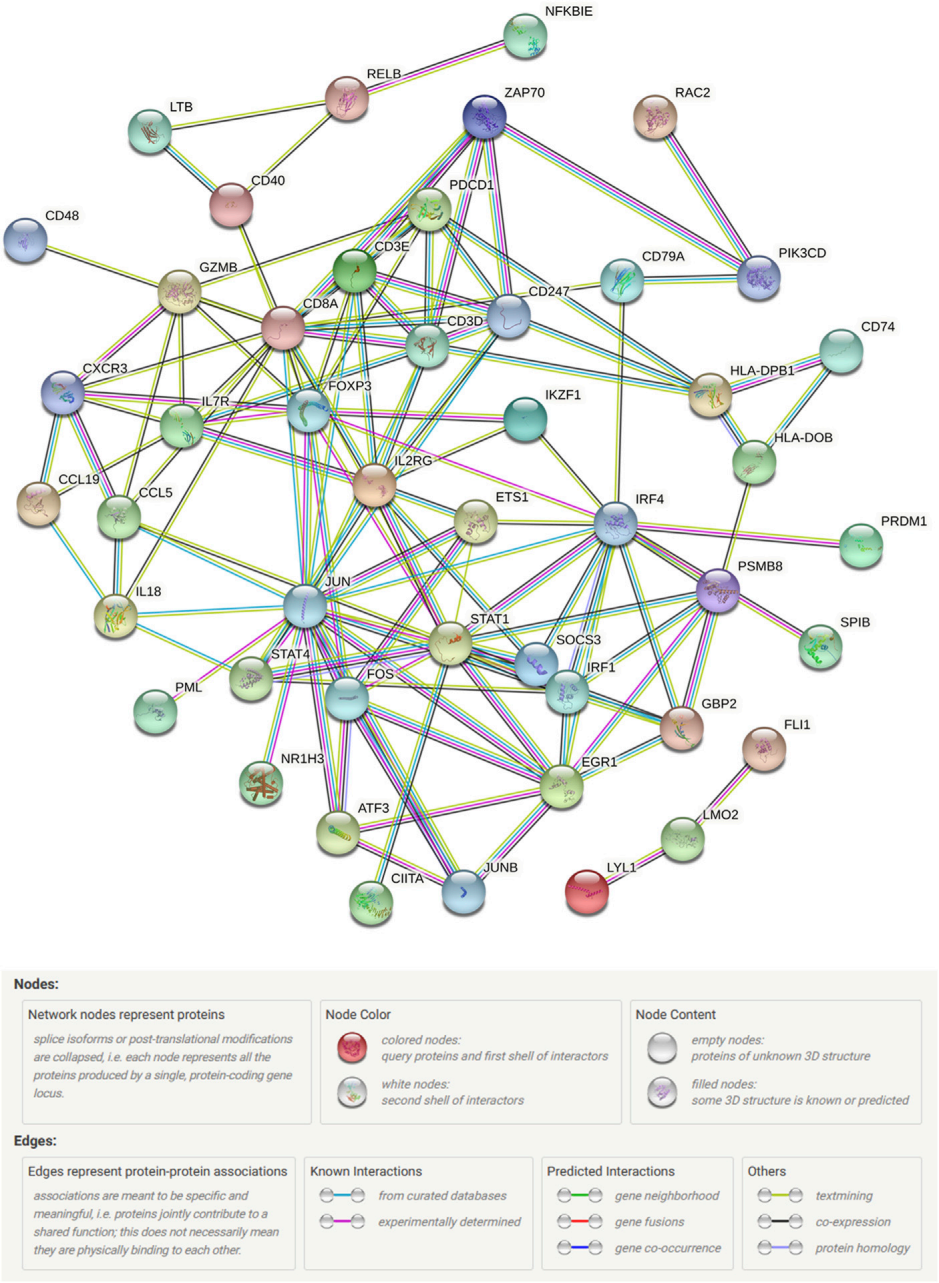
STRING protein–protein interaction network connectivity for prognostic immune-related genes and transcription factors.

Gene expression profiles of 139 breast cancer samples were downloaded from GSE45255. Expression levels of 259 out of 413 immune-related DEGs were shared by TCGA and GSE45255 databases. Based on the clinical data from TCGA database, 42 immune-related DEGs were found to be significantly related to OS by univariate Cox regression analysis, thereby considered as PIGs (Figure 5A). Following this, we downloaded 317 TFs from the Cistrome platform. The results of co-expression analyses revealed that 21 types of TFs, including CIITA, FOXP3, IKZF1, and IRF4, highly correlated with 35 types of PIGs (Figure 5B). Furthermore, the PPI network between the TFs and PIGs was generated by STRING, with FOXP3, JUN, STAT1, and CD3D at the center (Figure 6).

### 3.5 Construction and validation of an immune-related prognostic model

**FIGURE 7:**
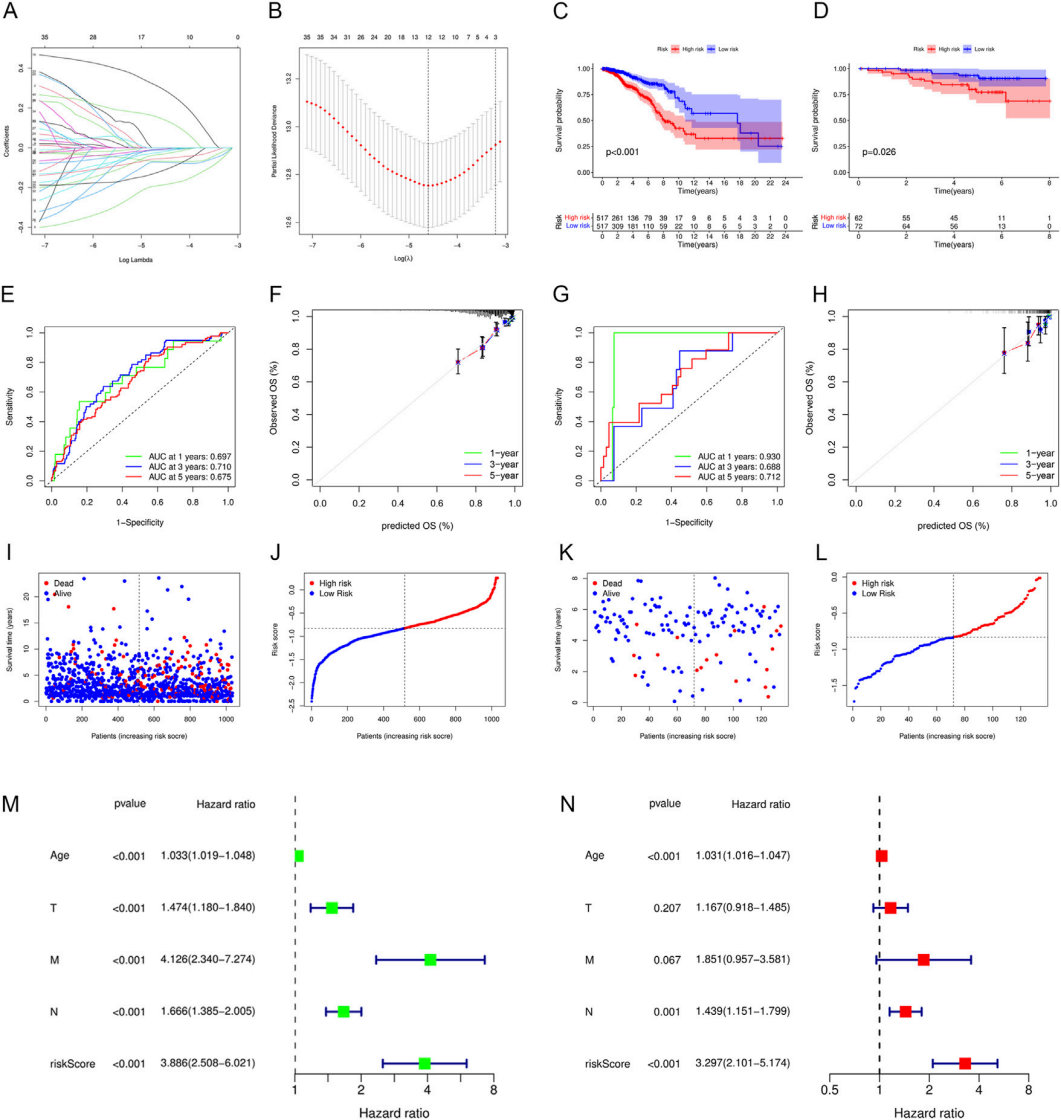
Construction and validation of an immune-related prognostic model. **(A)** Plot of LASSO coefficient profiles. Variables come to zero as we increase the penalty (lambda) in the objective function of LASSO. **(B)** Plot of partial likelihood deviance for 42 prognostic immune-related genes. Tenfold cross-validation was applied for turning parameter selection in the LASSO regression model, with 12 prognostic genes left for model construction. **(C,D)** Patients in the low-risk group showed better prognosis than those in the high-risk group for both **(C)** the training set and **(D)** validation set. **(E–H)** ROC and calibration curves for predicting the overall survival of the **(E,F)** training set and **(G,H)** validation set at 1, 3, and 5 years. **(I–L)** Distributions of risk scores and survival status of the **(I,J)** training set and **(K,L)** validation set. **(M,N)** Forest plots showing **(M)** univariate and **(N)** multivariate Cox regression results of the risk score and clinicopathologic factors.

In total, 12 out of 42 PIGs were chosen for model construction based on LASSO regression analysis: *TAPBPL*, *RAC2*, *IL27RA*, *ULBP2*, *PSMB8*, *SOCS3*, *NFKBIE*, *IGLV6-57*, *CXCL1*, *IGHD*, *AIMP1*, and *CXCL13* (Figures 7A,B). The coefficients for 12 PIGs were calculated through LASSO regression (Table 1). The validation set contained 134 samples from GSE45255, while the training set contained 1,034 samples from TCGA database. Each sample was assigned a risk score and categorized as either high risk or low risk. Results from both the training set and the validation set indicated that the low-risk group had a significantly better prognosis than the high-risk group (Figures 7C,D, *p* < .001 and *p* = .026 for the training set and the validation set, respectively). Figures 7E,F and Figures 7G,H depict the time-dependent ROC and calibration curves for training and validation sets, respectively. The AUCs (areas under curves) at 1, 3, and 5 years of this prognostic model were .697, .710, and .675 for the training set and .930, .688, and .712 for the validation set, respectively. In this model, the calibration curves for 1-, 3-, and 5-year survival probabilities corresponded well with the observed survival rates for both sets. The distributions of the risk scores, survival status, and survival time of the training and validation sets are plotted in Figures 7I–L. Moreover, univariate and multivariate Cox regression analyses validated the risk score as an independent prognostic factor after adjusting for age, T, M, and N stages (Figures 7M,N, *p* < .001).

**TABLE 1:** LASSO coefficient profiles of 12 prognostic immune-related genes.

Gene	Coef	Gene	Coef
*TAPBPL*	−.18851	*NFKBIE*	−.00698
*RAC2*	−.07299	*IGLV6-57*	−.00807
*IL27RA*	−.06814	*CXCL1*	−.11876
*ULBP2*	.301293	*IGHD*	−.05503
*PSMB8*	−.02251	*AIMP1*	.148086
*SOCS3*	−.0334	*CXCL13*	−.01237

### 3.6 Correlations between PIGs and immune infiltrates

**FIGURE 8:**
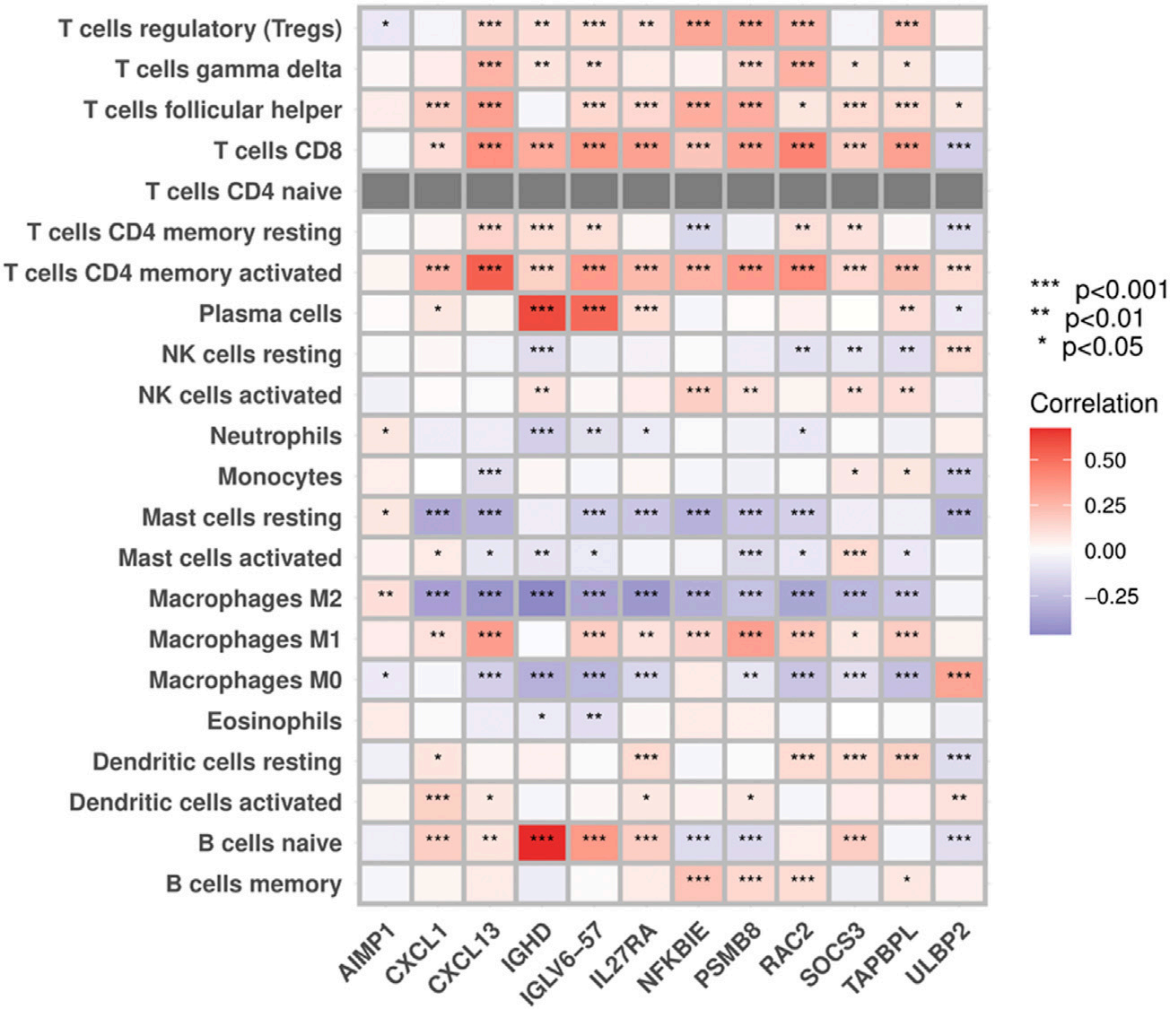
Correlations between prognostic immune-related genes and immune infiltrates.

Results from the “CIBERSORT” algorithm showed that except for AIMP1, which exhibited no significant correlation with most of the immune cell types, most PIGs in the model were positively associated with adaptive immune cells, such as T cells, B cells, and macrophages M1, and negatively associated with immune regulatory cells, such as macrophages M2, M0, and resting mast cells (Figure 8).

### 3.7 Correlations between breast cancer-related genes and the prognostic model

**FIGURE 9:**
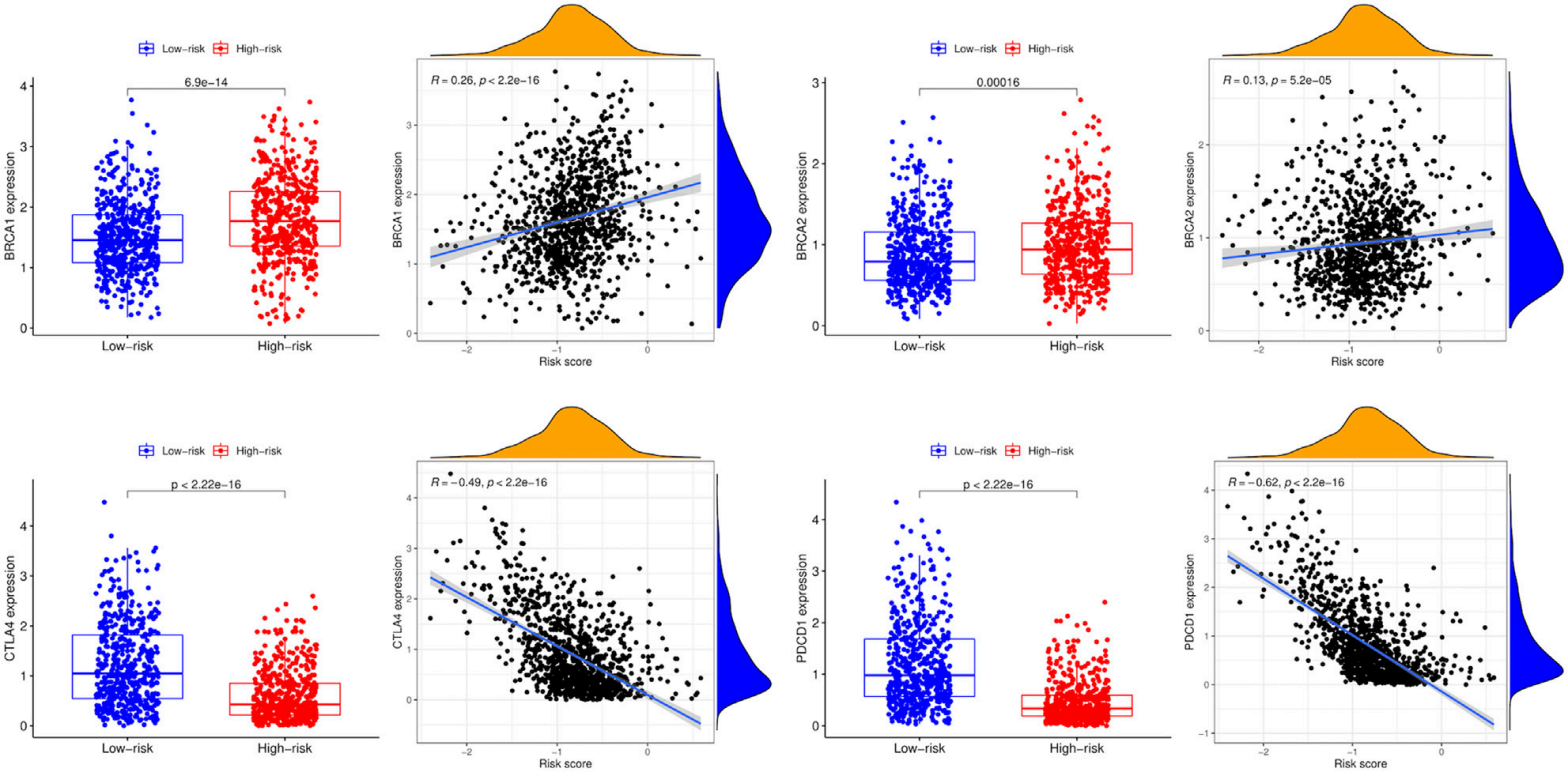
Correlations between breast cancer-related genes and the risk score.

Compared to the low-risk group, the expression levels of BRCA1 and BRCA2 were higher in the high-risk group, while CTLA4 and PDCD1 were lower. In addition, BRCA1 and BRCA2 expression levels were positively associated with the risk score, whereas CTLA4 and PDCD1 expression levels were negatively associated with the risk score (Figure 9).

### 3.8 TMB analysis

**FIGURE 10:**
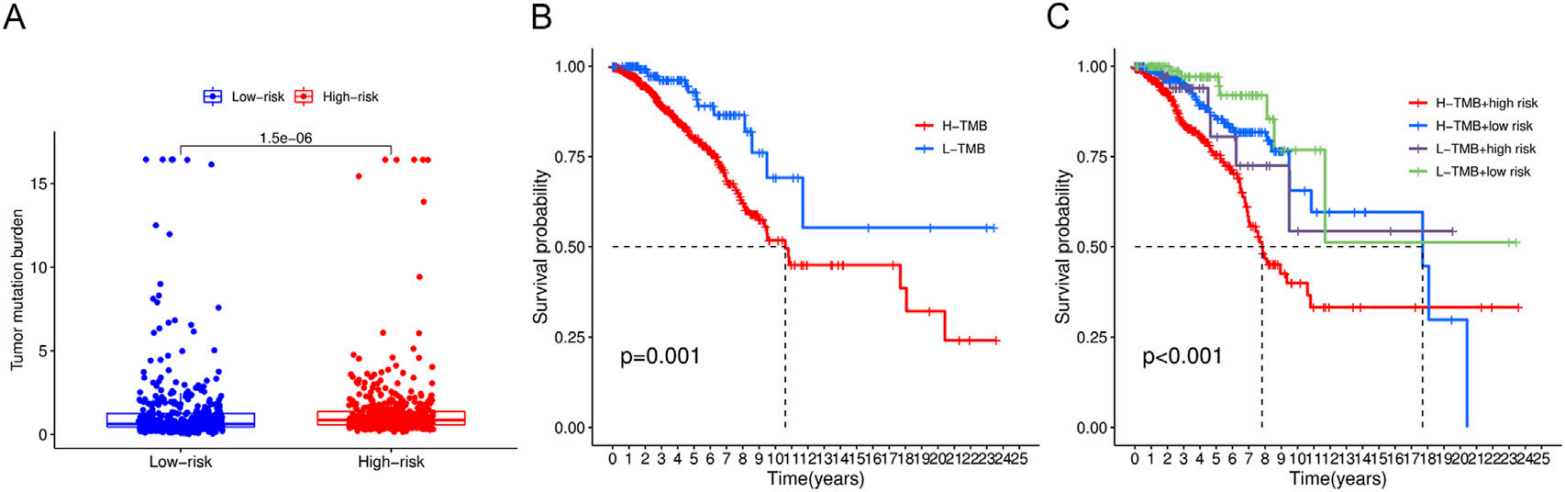
Tumor mutational burden analysis. **(A)** Boxplots demonstrated a higher TMB in the high-risk group. **(B)** Low-TMB group was associated with a favorable survival probability. **(C)** Patients in the low-TMB plus low-risk group had better prognosis than those in the high-TMB plus high-risk group.

TMB with a mean of 1.562 mut/Mb (range: .026–118,447 mut/Mb) was obtained from TCGA VarScan2 for 980 breast cancer samples. Figure 10A demonstrates that TMB was significantly higher in the high-risk group than in the low-risk group (*p* < .0001). Based on a cutoff value of 1 mut/Mb, patients were divided into high-TMB (*n* = 362) and low-TMB (*n* = 618) groups. The survival analysis revealed that the low-TMB group was associated with a longer OS than the high-TMB group (*p* = .001, Figure 10B). In addition, the OS of patients in the low-TMB and low-risk group was significantly superior to that of patients in the high-TMB and high-risk group (*p* < .001, Figure 10C).

### 3.9 Construction and validation of a nomogram

**FIGURE 11:**
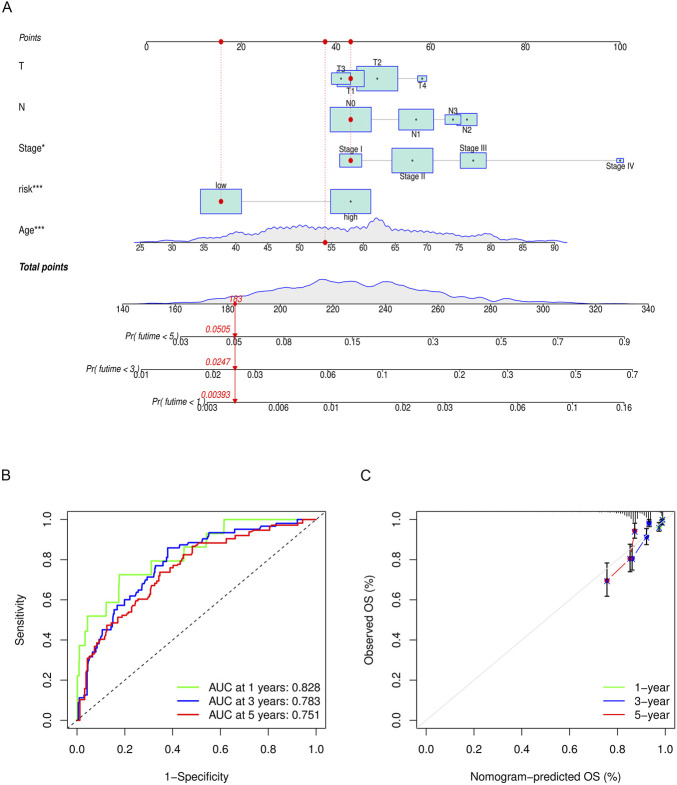
**(A)** Nomogram to predict the overall survival of breast cancer patients. The overall survival probability is calculated by taking the sum of the risk points, according to the T stage, N stage, clinical stage, risk group, and age. For each parameter, its risk point can be determined by drawing a vertical line straight up from the variables’ value to the “Points” axis. In order to determine the probability of surviving less than 5 years, a vertical line is drawn intersecting the “Total points” with the “Pr (futime< 5)” line. **(B)** 1-, 3-, and 5-year ROC curves of the nomogram. **(C)** 1-, 3-, and 5-year calibration curves of the nomogram.

To predict the 1-, 3-, and 5-year OS of breast cancer patients, a nomogram was constructed based on TCGA data. The T stage, N stage, clinical stage, risk group, and age were eventually utilized as parameters (Figure 11A). The M stage was excluded due to the imbalance of sample distribution. The 1-, 3-, and 5-year ROC curves of the nomogram were plotted, with respective AUCs of .828, .783, and .751 (Figure 11B). The calibration curve fitted well with the ideal model (Figure 11C).

## 4 Discussion

Breast cancer as a disease entity is characterized by vast heterogeneity. Beyond the current classification method based on pathology, gene expression profiling has subdivided breast cancer into subtypes with distinct biological behaviors. Intrinsic genomic, transcriptomic, and molecular complexities had a substantial influence on treatment response and prognosis (Prat et al., 2015). In recent years, open access to the next-generation sequencing data through public databases such as TCGA and GEO has allowed us to stratify risk based on the genomic heterogeneity of tumors. Using bioinformatics techniques, we built a risk model of 12 immune-related genes in this study. The 12 immune-related genes in the model demonstrated a strong correlation with breast cancer prognosis and immune infiltrates. Breast cancer samples in the low-risk group expressed higher levels of genes simulating the adaptive immune response and had a more favorable prognosis. The immune-related model could not only improve our ability to predict breast cancer patients’ prognosis but also help understand the immune mechanisms involved in tumorigenesis. Our study found that tumor samples in the high-risk group expressed higher levels of BRCA1 and BRCA2 but lower levels of CTLA4 and PDCD1. Intriguingly, a higher TMB was associated with the high-risk group. Incorporating parameters including T stage, N stage, age, and clinical stage, we developed a nomogram for predicting OS in patients with breast cancer. Both the risk model and nomogram showed good accuracy, reliability, and sensitivity in view of the ROC curve and calibration curve.

In an effort to identify PIGs to build the immune-related model, we first classified patients into clusters with high and low immune cell infiltration based on their ssGSEA immune enrichment scores. According to the GO annotation and KEGG enrichment results, DEGs of the high infiltration cluster were dominated by the cytokine receptor interaction signaling pathway and molecule of bacterial origin. Bioactive metabolites, such as reactivated estrogens, amino acid metabolites, short-chain fatty acids, and secondary bile acids, were secreted by microbiota and modulated tumor cell viability, migration, and apoptosis (Eyvazi et al., 2020). In recent years, accumulating evidence has demonstrated a close relationship between the intestinal bacterial microbiome and the progression and treatment of various tumors (Matson et al., 2021; Si et al., 2021; Wong-Rolle et al., 2021). For instance, enteric bacterial genes may metabolize estrogens and influence the incidence of ER-positive breast cancer (Kwa et al., 2016). Microbial perturbation was reported to contribute to epigenetic reprogramming and gene hyper-methylation in the development of breast cancer (Nagarajan and McArdle, 2018). The intricate interaction between pathogenic microbes and breast cancer cells warrants additional study.

Moreover, the PPI network uncovered the crucial roles of numerous proteins (e.g., FOXP3, STAT1, STAT4, FOXP3, JUN, and CD3D) in breast cancer. FOXP3 was reported to promote tumor growth and metastasis by activating the Wnt/β-catenin signaling pathway (Yang et al., 2017), according to previous research studies on non-small cell lung cancer. The STAT family consists of six isoforms, and the JAK-STAT pathways play varying roles in breast cancer progression and metastasis (Wong et al., 2022). The correlation between the associated signaling pathways and immunotherapy for breast cancer is anticipated to be investigated in future experiments.

In our research study, TMB showed a promising prognostic value for breast cancer patients, whether used alone or in conjunction with the risk score model. TMB, which is defined as the number of non-synonymous somatic mutations per MB of a cell’s genome, indirectly reflects heterogeneity and immunogenicity and predicts clinical response to immune checkpoint inhibitors in solid tumors such as melanoma, non-small-cell lung cancer, rectal cancer, and breast cancer (Snyder et al., 2014; Yarchoan et al., 2017). It was widely believed that TMB benefited immunotherapy because it could produce more antigens to simulate antitumor response (Rizvi et al., 2015). Data from the phase 3 KEYNOTE-119 study suggested the clinical benefits of pembrolizumab monotherapy but not chemotherapy in metastatic TNBC with TMB ≥10 mut/Mb (Winer et al., 2020). Also, according to results from the phase 2 TAPUR study, pembrolizumab monotherapy exerted antitumor activity in heavily pretreated metastatic breast cancer with high TMBs (9–37 mut/Mb) (Alva et al., 2021). However, seemingly inconsistent with previous findings, we found that a high TMB was associated with the high-risk group and a poor prognosis for breast cancer. The discrepancy of results could be explained in the following aspects. First, unlike the two clinical trials focusing on TMB ≥10 mut/Mb, only 1.5% (15/980) of our samples showed a TMB of over 10 mut/Mb. Second, this study focused on breast cancer in general rather than TNBC. In fact, TNBC was generally associated with a poor prognosis and harbored higher mutational rates than other subtypes of breast cancer (Kriegsmann et al., 2014). Another bioinformatics study pertaining to TNBC revealed a higher 5-year survival rate in the high-TMB group (Gao et al., 2021). Taken together, TMB may have disparate prognostic values among subgroups of breast cancer patients. For patients with non-TNBC, high levels of TMB are likely to suppress the immune response and reduce the survival rate. For patients with TNBC, survival benefits might only exist for those with high levels of TMB. In support of our speculation, the GeparNuevo trial reported a negative correlation of TMB with the frequency of CD8^+^ T effector cells, whereas a positive correlation with CD8^+^ T memory cells in the early-stage TNBC. The reported TMB (mean 1.8 mut/Mb and range .02–7.65 mut/Mb) was comparable to ours (Seliger et al., 2019). In support of to our speculation, the GeparNuevo trial reported a negative correlation of TMB with the frequency of CD8^+^ T effector cells, whereas a positive correlation with CD8^+^ T memory cells in the early-stage TNBC. Demonstrating unique immune characteristics of breast cancer and close relationships with TILs and TMB, our model could be utilized to predict the efficacy of therapies for TNBC patients.

The present study has limitations that should be carefully considered. First, we constructed the prognostic signature using the data downloaded from various publicly accessible databases. Publication bias and batch effect cannot be precisely measured, and additional research studies were necessary to validate the model. Second, genetic testing for genes of our model could be costly. Moreover, the prognostic model was developed using the data from a general breast cancer population, not a specific subtype, resulting in a limited ability to predict survival for breast cancer subtypes. Future evaluation on the correlation between TMB and prognosis of different breast cancer subtypes is anticipated to validate our findings and guide the application of immunotherapy. Last but not least, the five parameters on which we based the nomogram may not be optimal due to a limited number of factors available online. Given additional clinical characteristics, a more accurate model and a nomogram could be developed.

In conclusion, we developed a robust prognostic model aggregating 12 immune-related genes for risk stratification and a nomogram that could reliably predict OS for patients with breast cancer, which offers new insights into breast cancer immune cells and tumorigenesis.

## Data Availability

The original contributions presented in the study are included in the article/Supplementary Material; further inquiries can be directed to the corresponding authors.
